# Chemical Profiling, Sensory Qualities, and Bioactivities of Essential Oils Obtained from *Aloysia citrodora* and *Bursera graveolens* Ecuadorian Plants Against the Mosquito *Aedes albopictus* (Skuse) (Diptera: Culicidae)

**DOI:** 10.3390/insects16020202

**Published:** 2025-02-12

**Authors:** Prangthip Parichanon, Roberta Ascrizzi, Camilla Tani, Maria Cristina Echeverria, Sania Ortega Andrade, Hugo Paredes, Isabella Taglieri, Guido Flamini, Francesca Venturi, Barbara Conti

**Affiliations:** 1 Department of Agriculture, Food and Environment, University of Pisa, Via del Borghetto 80, 56126 Pisa, Italy; prangthip.parichanon@agr.unipi.it (P.P.); camilla.tani@phd.unipi.it (C.T.); isabella.taglieri@unipi.it (I.T.); francesca.venturi@unipi.it (F.V.); 2 Department of Pharmacy, University of Pisa, Via Bonanno 6, 56126 Pisa, Italy; roberta.ascrizzi@unipi.it (R.A.); guido.flamini@unipi.it (G.F.); 3 Nutrafood Research Center, University of Pisa, Via del Borghetto 80, 56124 Pisa, Italy; 4 eCIER Research Group, Department of Biotechnology, Universidad Técnica del Norte, Av. 17 de Julio 5–21 y Gral. José María Córdova, Ibarra 100150, Ecuador; mecheverria@utn.edu.ec (M.C.E.); smortega@utn.edu.ec (S.O.A.); hoparedes@utn.edu.ec (H.P.)

**Keywords:** Asian tiger mosquito, bio-based mosquito repellent, essential oil composition, lemon verbena, palo santo, smell profile

## Abstract

The mosquito *Aedes albopictus* is a vector of several harmful viruses, necessitating the development of safer and more sustainable repellents than DEET. This study evaluated the potential of plant-based EOs extracted via steam distillation from *Aloysia citrodora* and *Bursera graveolens* plants harvested in Ecuador. *A. citrodora* EO exhibited a high sensory pleasantness with a citral-like aroma, while *B. graveolens* EO had lower sensory appeal due to off-flavors. At 200 µL L^−1^, *B. graveolens* EO demonstrated strong ovideterrent activity, reducing egg laying by 64%, whereas *A. citrodora* EO displayed the highest larvicidal activity with an LC_50_ value of 88.54 µL L^−1^. Both EOs, applied at 0.20 µL cm^−2^ on human skin, provided comparable repellency for over 45 min. The RD_50_ values were 0.104 µL cm^−2^ for *A. citrodora* EO and 0.136 µL cm^−2^ for *B. graveolens* EO. When compared to DEET at 0.04 µL cm^−2^, no significant differences in repellency were observed within the first 15 min post-application. These findings suggest that both EOs are effective alternatives to synthetic repellents, and *A. citrodora* EO emerges as a promising candidate due to its superior sensory attributes and larvicidal activity.

## Simple Summary

Mosquitoes like the Asian tiger mosquito (*Aedes albopictus*) can spread dangerous diseases, making effective repellents essential. This study explored two aromatic plants, *Aloysia citrodora* and *Bursera graveolens*, as safer alternatives to synthetic repellents like DEET. These plant-based oils showed strong mosquito-repelling properties. The oil from *A. citrodora* was especially effective at killing mosquito larvae, while the oil from *B. graveolens* was excellent at preventing mosquitoes from laying eggs. Both oils provided protection similar to DEET for short periods, though they wore off faster because their natural ingredients break down quickly. Among the two, *A. citrodora* oil stood out for its pleasant citrus-like scent, making it a better choice for creating mosquito repellent products. With improved formulations to make them last longer, these natural oils could become effective, eco-friendly solutions for preventing mosquito bites and reducing disease risk.

## 1. Introduction

Climate change impacts the spread of vector-borne diseases by creating favorable conditions for climate-sensitive vectors like mosquitoes due to increased temperatures and altered rainfall patterns [1]. Throughout the past few decades, there has been an increase in the frequency, severity, and length of heat waves in Europe (EU), which has led to a worsening of exposure to these occurrences in particular in southern Europe [2]. Between 2011 and 2022, 11 EU countries reported summertime outbreaks of the West Nile virus (WNV) to the European Centre for Disease Prevention and Control (ECDC), which anticipates a yearly increase in reported cases due to changing environmental conditions [3].

*Aedes albopictus* (Skuse) (Diptera: Culicidae), often referred to as the Asian tiger mosquito, is a highly adaptable and invasive insect species that has spread globally, thriving even in temperate regions by enduring winter conditions. Numerous studies have demonstrated that it plays a key role in transmitting viruses like WNV, Chikungunya (CHIKV), Zika, and Dengue (DENV) across several regions, including central Africa, the Indian Ocean, and the EU [4]. As a result, *Ae. albopictus* populations, which thrive in highly urbanized environments, not only create an annoyance through their bites but also present considerable public health risks by transmitting various arboviral diseases to humans [5].

Several studies have reported that *Ae. albopictus* is an aggressive mosquito species due to its biting behavior, in particular during the early morning and late afternoon [6,7,8]. This mosquito possesses a swift and agile bite, enabling it to evade most human attempts to swat it. With no available vaccines or specific treatments for the primary diseases spread by *Ae. albopictus*, such as DENV and CHIKV, controlling the mosquito population is essential for disease prevention and management [9]. The prevailing methods for managing *Ae. albopictus* involve reducing larval populations by eliminating water-holding containers that serve as breeding grounds and using synthetic larvicides [10]. However, Paupy et al. [11] found that attempts to reduce mosquito larval breeding sites were ineffective in controlling the adult mosquito population due to the peculiar behavior of the species, which prefers to lay its eggs in small containers with very limited amounts of water on private property. Hence, utilizing repellents to safeguard individuals from mosquitoes has been demonstrated to be an advantageous strategy that can effectively mitigate the global transmission of numerous diseases carried by these insects [12].

Mosquito repellents are chemically volatile substances that, when applied to human skin, repel mosquitoes by driving them away from the source. This discourages mosquitoes from encountering the skin and prevents them from biting. Although synthetic mosquito repellents have been developed with the idea of consumer benefits, nowadays there is concern about the potential toxicity in terms of both human health and environmental problems caused by the massive use of these compounds [13]. These issues are now the primary driving force behind a quick search for novel insect repellents, derived from plant extracts, that are safe for the environment and human health by using them as alternative methods to protect humans from mosquito bites.

Essential oils (EOs) are volatile compounds responsible for the distinctive aromas of plants with their composition and bioactivity influenced by the growing environment [14,15]. Among the approximately 17,000 recognized aromatic plant species worldwide [16], *Aloysia citrodora* Paláu (Verbenaceae, commonly called lemon verbena) and *Bursera graveolens* (Burseraceae, known as Palo Santo) have been widely used for their medicinal properties across various cultures [17,18]. These plants are particularly valued for their insect-repelling effects [19,20]. Therefore, they have high potential as bio-based mosquito repellents. *A. citrodora*, a perennial shrub indigenous to South America, and *B. graveolens*, a deciduous tree found in dry forests from southern Mexico to northwest Peru, both cultivated across Andean nations, are widely used in traditional medicine in Ecuador and Peru [21]. To the best of our knowledge, nothing was reported on the repellent activity of *A. citrodora* EO and *B. graveolens* EO. Therefore, the objectives of this study were (i) to verify the bioactivity of the two EOs as larvicides and ovideterrents, (ii) to assess the pleasantness of the EOs for topical use by an expert panel, and (iii) to determine the efficacy and durability of the EOs in repelling *Ae. albopictus* females and comparing their repellence with that of DEET, which is the most potent repellent commercially available.

## 2. Materials and Methods

### 2.1. Botanical Sample Collection and Preservation

As described by Farina et al. [21], the botanical samples were collected in Ecuador with authorization from the Ecuadorian Ministry of Environment (authorization MAATE-ARSFC-2023-0036). Leaves of *A. citrodora* were sourced from the Intag Valley (0°18′15.00″ N, 78°34′27.00″ W), and stems of *B. graveolens* were collected from La Carolina parish (0°33′17.300″ N, 78°07′46.100″ W). The two regions are located in the province of Imbabura, Ecuador. These species are cultivated as part of an agroforestry system situated at altitudes of 900 to 1200 m above sea level. The region experiences annual rainfall of 1500–1750 mm and maintains an average temperature of 20–22 °C.

The samples were harvested without damaging the surrounding environment. They were immediately transported to the Department of Biotechnology at Universidad Técnica del Norte, Ibarra, Ecuador. They were stored at room temperature (~25 °C) in a light-protected environment before processing. Specific details regarding the collection protocols, preservation techniques, and sample handling are outlined in the methodology of Farina et al. [21].

### 2.2. Extraction Method of Botanical Essential Oils

The botanical samples were dried and ground into a fine powder. The hydrodistillation (HD) process for the extraction of EOs was conducted at the Department of Biotechnology, Universidad Técnica del Norte, Ibarra (Ecuador) as described by Farina et al. [21]. First, 100 g sample of each plant was air dried and was subjected to HD in a Clevenger-type apparatus for 2 h. The process was maintained at 110 °C to ensure constant boiling of the water to facilitate efficient extraction. The obtained EOs were placed in glass vials with anhydrous sodium sulfate to eliminate any residual moisture and then stored at a temperature of 4 °C until analysis.

### 2.3. GC-MS Profiling of A. citrodora and B. graveolens

Gas chromatography coupled with mass spectrometry (GC–MS) was conducted at the Department of Pharmacy, University of Pisa, Italy, to evaluate the chemical profiles of both EOs. A Varian CP-3800 gas chromatograph (Agilent Technologies Inc., Santa Clara, CA, USA) with an HP-5 capillary column (30 m × 0.25 mm, 0.25 µm coating) (Agilent Technologies Inc., Santa Clara, CA, USA) and a Varian Saturn 2000 ion trap mass detector (Agilent Technologies Inc., Santa Clara, CA, USA) was used. The EOs were diluted to 10% in HPLC-grade *n*-hexane before injection. The analytical conditions were set as reported in Farina et al. [21]. In detail, the injector and transfer line temperatures were 220 and 240 °C, respectively; the oven temperature was programmed at 60 to 240 °C, at 3 °C/min; the carrier gas was helium at 1 mL/min flow rate; the injection volume was 1 µL; the split ratio was 1:25. Data acquisition was performed in full scan mode, covering a mass range of 30 to 300 *m*/*z*, with a scan time of 1.0 s per cycle.

The individual compounds were identified by matching their mass spectra with those in the NIST 2017 mass spectral database accessed on 15 November 2024 [22]. Additionally, the retention indices (RIs) of the separated compounds were calculated using n-alkanes (C8–C20) as external standards and analyzed under the same GC-MS conditions as those used for the EO samples to ensure consistency in the analysis. These RI values were compared with published data to verify the compound identify and elution order [23].

### 2.4. Sensory Assessment

Eight trained experts from the Department of Agriculture, Food, and Environment (DAFE) at the University of Pisa, with prior experience in sensory descriptive analysis [24,25], evaluated the aromatic profiles of *A. citrodora* and *B. graveolens* EOs using a custom sensory form with a 0–9 scale for “intensity”, “persistence”, and “pleasantness”. Panelists also suggested olfactory descriptors. The evaluations took place in a quiet, well-ventilated room, with each panelist receiving a scent strip infused with 10 μL of anonymized EO, and a 15-min interval between assessments to avoid cross-contamination.

### 2.5. Rearing of Ae. Albopictus Mosquitoes

*Ae. albopictus* samples were collected from wild eggs using ovitraps (Entomox srl., Pisa, Italy) placed in the DAFE gardens (latitude 43°42′48″ N, longitude 10°24′52″ E), which were areas with high mosquito activity as identified in previous inspections. The ovitraps consisted of plastic black pots filled with tap water and provided with four brown masonite strips (ovistrips) for egg collection as seen in Müller et al. [24]. The mosquitoes were reared under laboratory conditions following the methods of Bedini et al. [25] and Najar et al. [26]. Newly emerged larvae were raised in plastic trays, fed cat food, and allowed to reach the pupal stage. About 300 pupae (with an equal male-to-female ratio) were placed into cylindrical Plexiglas cages (35 cm in diameter and 60 cm in length) and covered with a cotton stockinet sleeve at the front to allow easy handling. The cages were kept under laboratory controlled conditions, and adult mosquitoes were given a 20% sucrose solution for feeding [26].

### 2.6. Larvicidal Effect

Bioassay tests were conducted in accordance with the World Health Organization’s standard method [27]. Ten freshly hatched fourth-instar larvae (0–24 h old) were transferred into beakers containing 250 mL of aqueous solutions of Tween 80 at 0.1% concentration with varying concentrations of EOs (50–600 µL L^−1^). A control group, consisting of ten larvae, was maintained in 0.1% Tween 80 solution without EOs. The bioassays, with four replicates per concentration (totaling 40 larvae per concentration), were conducted under standard laboratory conditions of 25 ± 2 °C, 65 ± 5% relative humidity (RH) and a photoperiod L:D = 12:12. No food was provided during the experiment, and mortality was recorded after 24 h from the beginning of the experiment (when the larvae were introduced into the solution) [25]. Abbott’s formula [28] was applied to adjust the mortality rates.

### 2.7. Oviposition Deterrence Assays

The ovideterrence activity of *A. citrodora* and *B. graveolens* EOs was assessed as described by Bedini et al. [25]. The experiment (June–September 2021) was conducted outdoors in a 3000 m^2^ garden at DAFE. Four ovitraps, as described earlier, were used to test the EOs at a concentration of 200 µL L^−1^ in a 0.1% Tween 80 water solution with each treated trap paired with a control containing only the 0.1% Tween 80 solution. Paired ovitraps were placed 5 m apart. Masonite strips (3 × 15 cm) were collected daily for 14 days. The number of *Ae. albopictus* eggs was counted under a stereo microscope, and the experiment was conducted in triplicate. Oviposition activity was assessed using the oviposition activity index (OAI):

OAI = (NT − NS)/(NT + NS)(1)

where NT is the total number of eggs in the test solution and NS is the total number of eggs in the control solution. Negative OAI values (<−0.3) indicated deterrence, while positive values (>+0.3) indicated attraction [29].

Effective repellency (%ER) was calculated as

%ER = [(NS − NT)/NS] × 100(2)

### 2.8. Protection Efficacy and Protection Time to Humans

The repellency of *A. citrodora* and *B. graveolens* EOs against *Ae. albopictus* adults was assessed using the human-bait technique (modified WHO protocol, 2009) [25]. The study, approved by the University of Pisa Ethics Committee (No. 32/2022), involved 10 non-allergic volunteers who provided written informed consent. Tests were conducted in the summer (9–11 a.m.) using 8–12-day-old, sugar-fed, blood-starved mosquitoes. Volunteers applied 100 μL of ethanol (control) or EO solution (0.04–0.20 µL cm^−2^) to a 25 cm^2^ skin area, and their hands were exposed to approximately 150 female mosquitoes for 3 min. Mosquito landings were recorded. The percentage of protective efficacy was calculated as

%PE = [(NPC − NPT)/NPC] × 100(3)

where NPC and NPT are the number of mosquito landings on the control and treated hands, respectively [30]. The repellency dosage values for 50% (RD_50_) were determined using log-probit regressions at EO doses of 0.004–0.40 µL cm^−2^ of skin.

Protection time was assessed by applying EOs at 0.02 µL cm^−2^ to the skin, which offered complete protection, and exposing it until two landings occurred during a single exposure or one landing was observed in two consecutive exposures. Complete protection time (CPT) was documented in accordance with Control of Neglected Tropical Diseases guidelines [31]. Four cages were randomly assigned and used in turn by each participant to avoid bias from repellent buildup in a single cage. The results of the CPT were compared with the ones obtained from the use of an ethanolic solution of DEET (Sigma-Aldrich, Milan, Italy) at the dose of 0.04 µL cm^−2^ of skin, serving as the positive control, while 100 µL of ethanol was used as the negative control.

### 2.9. Statistical Analysis

Big Sensory Soft 2.0 (version 2018; Centro Studi Assaggiatori, Brescia, Italy) was used to process sensory analysis data. Median values were analyzed using Friedman analysis of variance (ANOVA). Variations in *Ae. albopictus* oviposition were reported as mean ± standard deviation (*n* = 12) and examined through one-way ANOVA with OAI as the dependent variable. Duncan’s post hoc test (*p* < 0.05) was applied for mean comparisons with OAI data subjected to arcsine transformation prior to analysis.

Probit regression was employed to determine the lethal concentration values for 50% (LC_50_) and 90% (LC_90_) mortality as well as the RD_50_ and the repellency dosage values for 90% (RD_90_), for larvicidal and repellent activities. Relative median potency (RMP) was used to evaluate differences between these values. One-way ANOVA, followed by Duncan’s post hoc test, was performed to analyze the repellency of EOs. Kaplan–Meier survival analysis was applied to estimate the median CPT along with the 95% confidence interval. All statistical tests were conducted using SPSS 22.0 (SPSS Inc., Chicago, IL, USA).

## 3. Results

### 3.1. EOs Compositions

The complete composition of both the studied EOs was already reported in Farina et al. [21]. The EOs yield was 0.18% (*w*/*w*) for *A. citrodora* and 1.35% (*w*/*w*) for *B. graveolens*, which was calculated based on the dry plant material.

For *A. citrodora* EO, 40 compounds were identified. Over 55% of the composition consisted of oxygenated monoterpenes, with geranial (26.8%) and neral (21.0%) being the most abundant, both contributing to a citrusy aroma [32]. This citrus aroma was also present in limonene (7.2%) and was classified as monoterpene hydrocarbons. Sesquiterpene hydrocarbons accounted for 18.1%, and bicyclogermacrene (6.8%) was the most represented compound, contributing a “green” odor note [32].

For *B. graveolens* EO, 23 compounds were identified with monoterpenes constituting over 80% of the composition. Among the monoterpene hydrocarbons, limonene was the most abundant compound, making up 46.2% of the total composition and contributing a citrusy aroma [32]. Oxygenated monoterpenes accounted for 34.6%, and α-terpineol (17.8%) was the second most abundant compound, which was characterized by a terpenic odor note [32].

### 3.2. EOs Smell Characterization

**Figure 1:**
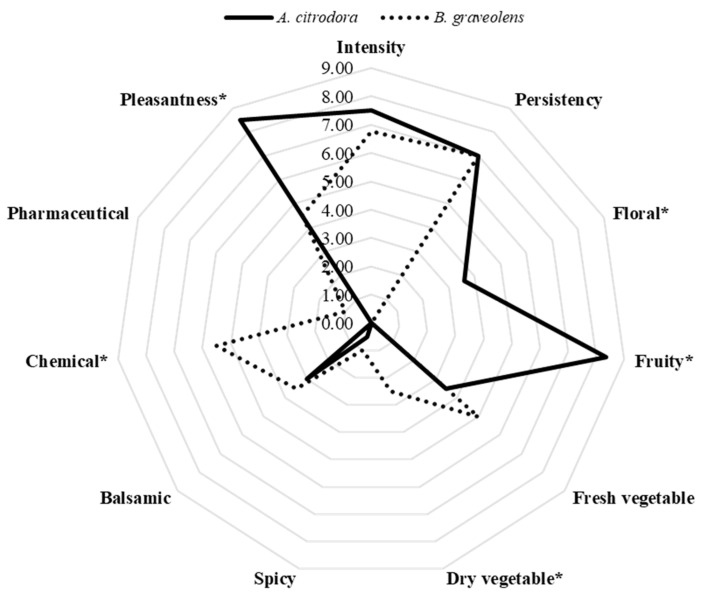
Complete organoleptic profiles based on median values. Asterisks indicate differences statistically significant between samples according to ANOVA of Friedman.

The two selected EOs showed the same “smell intensity” and “olfactory persistence”, while they differed significantly from each other in terms of specific smell profiles and “pleasantness” (Figure 1).

As reported in Figure 1 and Table 1, the best smell profile was described for *A. citrodora* EO, which was based on the highest values for positive notes such as fruity (mainly citrus notes of lemon, candied lemon, and lemongrass) and floral (mainly orange blossom). On the contrary, *B. graveolens* was characterized by the lowest smell pleasantness (Figure 1), due to the highest number of off-flavors (Table 1) indicated by panelists during assessment (i.e., mold, swamp, backwater, paint, etc.).

### 3.3. EOs Oviposition Deterrence

In our field experiments, the type of EO significantly influenced the oviposition deterrence of *Ae. albopictus* females, as shown in Table 2. At a concentration of 200 µL L^−1^, *B. graveolens* EO exhibited a stronger deterrent effect (ER: 63.69 ± 10.07%, OAI: −0.47 ± 0.11) compared to *A. citrodora* EO (ER: 31.97 ± 8.72%, OAI: −0.19 ± 0.06). However, the total number of eggs laid in traps treated with *B. graveolens* EO (363 eggs) was higher than those treated with *A. citrodora* EO (235 eggs). This discrepancy may be explained by the control traps, where the oviposition activity was significantly higher for the control traps paired with *B. graveolens* EO (993 eggs) than with the ones paired with *A. citrodora* EO (344 eggs).

### 3.4. EOs Larvicidal Effect

Table 3 shows that *A. citrodora* and *B. graveolens* EOs can act as larvicides against Asian tiger mosquito fourth instar larvae. The most potent, after 24 h of treatment, was *A. citrodora* EO, with an LC_50_ and an LC_90_ of 88.5 µL L^−1^ and 131.446 µL L^−1^, respectively. For *A. citrodora* EO, since its LC_50_ value was less than 100 µL L^−1^, it can be classified as strong larvicidal against *Ae. albopictus* [33].

### 3.5. EOs Repellent Activity

To our knowledge, there are no data in the literature on the repellent activity of *A. citrodora* and *B. graveolens* EOs for topical application against females of *Ae. albopictus*. This study evaluated the mosquito-repellent effectiveness of both EOs in vivo through the human-arm-in-cage test, following the WHO 2009 guidelines. Table 4 shows the repellency of *A. citrodora* and *B. graveolens* EOs at the different concentrations for 45 min. The results are expressed as a percentage of protection efficacy. The results revealed that *A. citrodora* EO protected completely against mosquitoes bites at all doses (0.04, 0.12, and 0.20 µL cm^−2^ of skin) for 5 min, while *B. graveolens* was less effective in protecting the skin from mosquitoes’ bites at the lowest concentrations (repellency of 90.55 ± 7.85% at 0.04 µL cm^−2^ of skin and 92.54 ± 3.01% at 0.12 µL cm^−2^ of skin). After 45 min, *A. citrodora* and *B. graveolens* EOs at the dose of 0.2 µL cm^−2^ of skin showed about the same percentage of protection efficacy at 68.30 ± 3.80% and 63.98 ± 2.05%, respectively, with no significant difference (*p* > 0.05).

The RD_50_ and RD_90_ values, representing the concentrations of both EOs required to repel 50% and 90% of the mosquitoes, respectively, are presented in Table 5. For both Eos, the results were very similar (RD_50_ of 0.140 and 0.136 µL cm^−2^ of skin for *A. citrodora* and for *B. graveolens* EOs, respectively). In our experiments, the CPT of the two EOs at the lowest doses (0.04 µL EO cm^−2^ of skin) showing 100% of PE was compared to the one of DEET (at the same dose), which is a commercially effective insect repellent.

**Figure 2:**
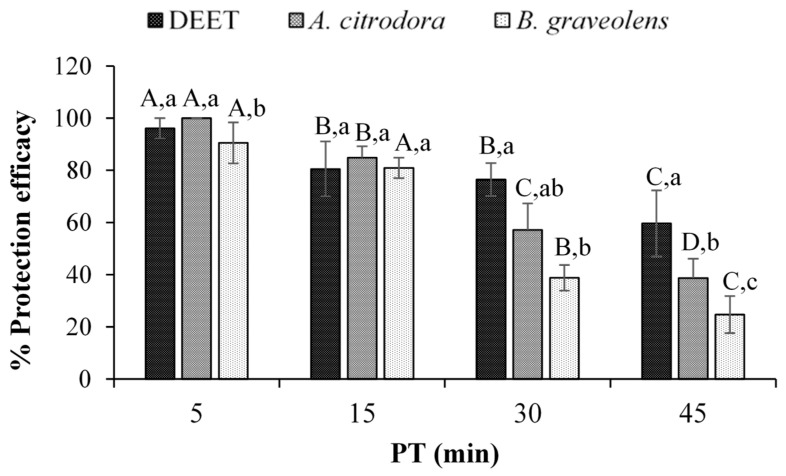
Protection efficacy (%) of the tested essential oils (*Aloysia citrodora* and *Bursera graveolens*) and DEET at a dosage of 0.04 µL cm^−2^ of skin against female *Aedes albopictus*. ^a–c^ Different lower-case letters indicate significant differences among treatments within the same time point (Duncan’s MRT, *p* ≤ 0.05). ^A–D^ Different upper-case letters indicate significant differences among time points within the same treatment (Duncan’s MRT, *p* ≤ 0.05). Error bars on each column represent standard deviation where *n* = 8.

From the results, shown in Figure 2, we deducted that at the beginning of the bioassay and up to 15 min after treatment, both EOs and DEET have similar repellency with no statistically significant variations (*p* > 0.05). Even if after 45 min, all the tested substances had a decreasing protection efficacy, DEET had the statistically highest percentage of protection efficacy (59.64 ± 12.67%, *p* < 0.05) compared to the EOs, but in any case, it was not useful to completely protect the skin. Both EOs exhibited similar levels of protective efficacy, and the difference between the two was not statistically significant (*p* > 0.05).

## 4. Discussion

An increasing number of individuals are opting for plant-based repellents due to the abundance of insecticidal secondary metabolites in these plants, which are generally safe and break down into non-toxic chemicals in humans [34]. The present study confirmed that EOs contain aromatic components that can be used against *Ae. albopictus*, providing both effective repellency and pleasant scents. The biological activity of various EOs can vary depending on the plant species, its origin, and its chemical composition [35]. This study found that both *A. citrodora* and *B. graveolens* EOs can effectively repel *Ae. albopictus*, reducing the percentage of eggs laid and acting as larvicides.

As previously described, limonene and α-terpineol were the primary components of *B. graveolens* EO, which was entirely composed of monoterpenes, predominantly in hydrocarbon form. The presence of limonene as a major component aligns with the findings of Jumbo et al. [20] and Monzote et al. [36], although their investigations did not reveal significant amounts of α-terpineol. In contrast, this oxygenated monoterpene was abundantly present in the EO derived from the stems of *B. graveolens* plants grown in Manabí (Ecuador), as studied by Fon-Fay et al. [37]. Meanwhile, the oxygenated monoterpenes geranial and neral were the major compounds in *A. citrodora* EO in the present study. This EO also contained notable amounts of monoterpene and sesquiterpene hydrocarbons, primarily limonene and bicyclogermacrene. These key components of *A. citrodora* EO align with the findings of Fitsiou et al. [38], who, however, reported an opposite ratio of the two isomers with a predominance of neral over geranial. Interestingly, Argyropoulou et al. [39] highlighted a strong influence of the phenological stage on geranial and neral, reporting greater percentages of the former during the vegetative stage and of the latter during the bloom stage.

Both *A. citrodora* and *B. graveolens* EOs showed mosquito repellency comparable to DEET during the initial 15 min of the bioassay, demonstrating their potential as effective natural alternatives for short-term applications. However, as the test progressed, the protective efficacy of DEET remained higher than that of both EOs, which gradually decreased over time. Despite this, the two EOs showed comparable levels of repellency, suggesting similar modes of action or effectiveness in repelling mosquitoes. In this investigation, limonene and α-terpineol were the main components of *B. graveolens* EO, while geranial and neral dominated *A. citrodora* EO. Andrade-Ochoa et al. [40] found that limonene at 0.02% can repel gravid females of *Culex quinquefasciatus* from laying eggs with 100% efficacy. These results support our findings that *B. graveolens* EO has better potential to repel *Ae. albopictus* females from laying eggs because limonene (46.2%) is its main component. However, despite its stronger deterrent effect, the overall number of eggs laid in traps treated with *B. graveolens* EO was higher than in those treated with *A. citrodora* EO. These findings suggest that environmental factors or the inherent attractiveness of volatile compounds in *B. graveolens* EO may have contributed to the observed total oviposition activity.

Regarding larvicidal activity, Giatropoulos et al. [41] found that lemon oil, primarily composed of limonene, γ-terpinene, neral, and geranial, was the most effective larvicidal agent. This finding aligns with our results, as *A. citrodora* EO demonstrated the strongest larvicidal activity. Our results also showed that both *A. citrodoraxc* and *B. graveolens* EOs could protect human skin from *Ae. albopictus*. This effect appears to be related to the high levels of monoterpenes [42]. In addition, Hao et al. [43] reported that adult *Ae. albopictus* were less likely to seek hosts and feed on blood when to vapors of geraniol, citral, eugenol, or anisaldehyde. Therefore, chemical repellents interact with odorant receptors in insects’ olfactory systems, altering their behavior and making it harder for them to approach their hosts.

In recent decades, growing attention has been directed toward EOs for mosquito prevention. Their increasing popularity can be attributed to consumers’ perception of these products as being safe [44,45]. EOs are often characterized by low toxicity toward humans; however, to be acceptable, an EO used for pest control must not be toxic to non-target organisms while remaining highly toxic to the targeted pests [46]. Both the EOs tested in the present study, as well as their major constituents, are listed as food additives permitted for direct addition to food for human consumption or are generally recognized as safe (GRAS) by the Food and Drug Administration (21 CFR Parts 170-186 [47]). As a result, they may represent valuable, eco-friendly alternatives for pest management. In our research, we found that the EOs from *A. citrodora* and *B. graveolens* had the same statistically repellent activity (with *A. citrodora* EO as the most repellent) against the Asian tiger mosquito for 15 min as DEET at the same dosage (0.04 µL cm^−2^ of skin). As DEET is the most effective repellent on the market, this comparison provides an excellent assessment of the performance of the two tested EOs. However, at the end of the test, the repellency of DEET was statistically higher than that of the EOs, which was likely because the volatile components of the EOs are more susceptible to degradation, oxidation and polymerization processes in the air, which can lead to a loss of efficacy over time. In contrast, DEET is known to be more stable due to its lower volatility [48]. This difference highlights the challenge of using EOs as repellents, as their volatile components can degrade more quickly than synthetic repellent DEET [49]. Therefore, formulation interventions such as the addition of volatile component stabilizers, nanoformulation or microencapsulation would be required to slow their volatility. Additionally, our findings indicated that *A. citrodora* EO received a notably high pleasantness score during the panel test. This was attributed to its sweet floral, rose, citrus, and fruity aromas, which were primarily derived from its major components, particularly the citral note. These results suggest that this EO could be a promising candidate for commercialization with appropriate formulation strategies [50].

## 5. Conclusions

This study highlights the potential of *A. citrodora* and *B. graveolens* EOs as eco-friendly and effective alternatives to synthetic repellents like DEET for the control of *Ae. albopictus* (Asian tiger mosquito). Both EOs exhibited significant repellency, larvicidal activity, and egg-laying deterrence against *Ae. albopictus*. Notably, *A. citrodora* EO, dominated by geranial and neral, showed superior larvicidal efficacy, while *B. graveolens* EO, rich in limonene and α-terpineol, demonstrated strong oviposition deterrence.

The repellency of these EOs was statistically comparable to DEET at the same concentration for a short duration, though their effectiveness diminished over time due to the volatility and oxidative degradation of their components. This limitation underscores the need for formulation enhancements, such as nanoencapsulation or the addition of stabilizers, to extend their efficacy.

Furthermore, *A. citrodora* EO received high sensory acceptability due to its pleasant sweet, floral, and citrus aromas, making it a promising candidate for commercialization. With appropriate formulation strategies, these EOs could serve as safe, sustainable, and effective repellents, catering to the growing demand for natural mosquito prevention solutions.

## Figures and Tables

**Table 1:** Specific descriptors for smell characterization as indicated by panelists during assessments.

Main Odor	*A.* *citrodora*	*B. graveolens*
Positive Attributes		
Fruity	Lemon, lemongrass	n.d.
Floral	Orange blossom	n.d.
Balsamic	Mint, menthol	n.d.
Spicy	Sweet pastry, candied lemon, candied orange	n.d.
Off-flavors		
Chemical	n.d.	Mold, backwater, swamp
Pharmaceutical	n.d.	Carbolic acid, paint

n.d. = not detected.

**Table 2:** Oviposition deterrent effect of *A. citrodora* EO and *B. graveolens* EO at the concentration of 200 µL L^−1^ against *Ae. albopictus* females. Each mean value was calculated with data recorded daily along a week on three replicates.

Essential Oil (EO)	Total No. of Eggs Laid		Average No. of Eggs/Ovitrap		% ER	OAI
	Control	Treated	Control	Treated		
*A. citrodora*	344	235	68.80 ± 19.02 ^b^	47.00 ± 13.95 ^a^	31.97 ± 8.72 ^b^	−0.19 ± 0.06 ^b^
*B. graveolens*	993	363	198.60 ± 14.31 ^a^	72.60 ± 23.16 ^a^	63.69 ± 10.07 ^a^	−0.47 ± 0.11 ^a^

In the No. of eggs/ovitrap, ER (%), and OAI column, different letters indicate significant differences (independent sample *t*-test, Sig. (2-tailed) < 0.05). Means were followed by standard errors. ER (%) percent effective repellence, OAI oviposition activity index.

**Table 3:** Larvicidal activity of *Aloysia citrodora* and *Bursera graveolens* essential oils.

	*A. citrodora*	*B. graveolens*
LC_50_^a^ (CI)	88.543 (79.384–96.302)	146.528 (138.427–154.942)
LC_90_^b^ (CI)	131.446 (121.157–145.647)	208.464 (191.457–237.813)
Slope ± SD	7.468 ± 0.896	8.370 ± 1.075
Intercept ± SD	−14.542 ± 1.833	−18.129 ± 2.333
χ^2^ (df)	0.754 (4)	2.125 (4)
*P*	0.944	0.713

^a^ Concentration of the EO that kills 50% of the exposed larvae; ^b^ Concentration of the EO that kills 90% of the exposed larvae. Data are expressed as μL L^−1^; CI, confidence interval; (df), degrees of freedom; *P*, Pearson goodness-of-fit test.

**Table 4:** Efficacy protection of *Aloysia citrodora* and *Bursera graveolens* essential oils at different dosages against *Ae. albopictus* during 45 min of observations.

Essential Oil	Dose (µL of EO cm^−2^ of Skin)	Protection Efficacy ± SD (%)				
		Time After the Application of the Repellent (min)				
		0	5	15	30	45
*A. citrodora*	0.04	100 ^A,a^	100 ^A,a^	84.87 ± 4.37 ^B,cd^	57.13 ± 10.19 ^C,b^	38.61 ± 7.44 ^D,c^
	0.12	100 ^A,a^	100 ^A,a^	89.32 ± 6.65 ^B,abc^	68.35 ± 6.94 ^C,a^	46.92 ± 0.67 ^D,b^
	0.20	100 ^A,a^	100 ^A,a^	94.83 ± 1.25 ^B,a^	73.91 ± 1.12 ^C,a^	68.30 ± 3.80 ^D,a^
*B. graveolens*	0.04	100 ^A,a^	90.55 ± 7.85 ^B,b^	80.89 ± 3.94 ^B,d^	38.77 ± 4.90 ^C,c^	24.73 ± 7.10 ^D,d^
	0.12	100 ^A,a^	92.54 ± 3.01 ^B,b^	86.87 ± 5.26 ^B,bcd^	50.74 ± 3.60 ^C,b^	40.01 ± 6.37 ^C,bc^
	0.20	100 ^A,a^	100 ^A,a^	92.00 ± 7.67 ^B,ab^	72.61 ± 7.67 ^C,a^	63.98 ± 2.05 ^C,a^
DEET	0.04	100 ^A,a^	96.15 ± 3.85 ^A,a^	80.54 ± 10.54 ^B,d^	76.41 ± 6.34 ^B,a^	59.64 ± 12.67 ^C,a^

The data represent the mean of eight replicates. Means followed by different letters are significantly different (Duncan’s MRT, *p* ≤ 0.05). At T = 0, all treatments exhibited 100% protection efficacy, indicating equal initial repellency before differences emerged over time. ^A–D^ Different superscript uppercase letters indicate significant differences within rows (Duncan’s MRT, *p* ≤ 0.05). ^a–d^ Different superscript uppercase letters indicate significant differences within columns (Duncan’s MRT, *p* ≤ 0.05).

**Table 5:** Repellent activity of *Aloysia citrodora*, *Bursera graveolens* essential oils, and DEET.

	*A.* *citrodora*	*B. graveolens*	DEET
RD_50_^a^ (CI)	0.104 (0.079–0.140)	0.136 (0.107–0.189)	0.035 (0.022–0.046)
RD_90_^b^ (CI)	1.057 (0.521–5.185)	1.107 (0.566–4.555)	0.200 (0.157–0.282)
Slope ± SD	1.273± 0.258	1.408 ± 0.265	1.683 ± 0.224
Intercept ± SD	1.251± 0.268	1.219 ± 0.271	2.457 ± 0.238
χ^2^ (df)	2.495 (1)	3.063 (1)	0.860 (2)
*P*	0.114	0.080	0.651

^a^ Concentration of the EO that repels 50% of the *Ae. albopictus* when compared to untreated control; ^b^ Concentration of the EO that repels 90% of the *Ae. albopictus* after 45 min exposure. Data are expressed as μL cm^−2^ of skin; CI, confidence interval; (df), degrees of freedom; *P*, Pearson goodness-of-fit test.

## Data Availability

The datasets are available from the corresponding author on request.

## Abbreviations

The following abbreviations are used in this manuscript:

ANOVA	analysis of variance
CHIKV	Chikungunya Virus
CPT	complete protection time
GC-MS	gas chromatography–mass spectrophotometry
DAFE	Department of Agricultural, Food, and Environment
DENV	Dengue Virus
ECDC	European Centre for Disease Prevention and Control
EOs	essential oils
EU	Europe
WNV	West Nile virus
OAI	oviposition activity index
CI	confidence interval
ER	effective repellency
HD	hydrodistillation
LC_50_	lethal concentration values for 50%
LC_90_	lethal concentration values for 90%
PE	protective efficacy
PT	protection time
RD_50_	repellent dosage values for 50%
RD_90_	repellent dosage values for 90%
RI	retention indices
RH	relative humidity
RMP	relative median potency
